# Adiponectin aggravates bone erosion by promoting osteopontin production in synovial tissue of rheumatoid arthritis

**DOI:** 10.1186/s13075-018-1526-y

**Published:** 2018-02-08

**Authors:** Jie Qian, Lingxiao Xu, Xiaoxuan Sun, Yani Wang, Wenhua Xuan, Qian Zhang, Pengfei Zhao, Qin Wu, Rui Liu, Nan Che, Fang Wang, Wenfeng Tan, Miaojia Zhang

**Affiliations:** 1 Department of Rheumatology, The First Affiliated Hospital of Nanjing Medical University, 300 Guangzhou Road, Nanjing, 210029 China; 2 Department of Rheumatology, Affiliated Hospital of Nantong University, 20 Xisi Road, Nantong, 226001 China; 3 Department of Cardiology, The First Affiliated Hospital of Nanjing Medical University, 300 Guangzhou Road, Nanjing, 210029 China

**Keywords:** Adiponectin, Bone, Erosion, Osteoclasts, Osteopontin, Rheumatoid arthritis

## Abstract

**Background:**

We have previously reported that adiponectin (AD), an adipokine that is secreted by adipocytes, correlates well with progressive bone erosion in rheumatoid arthritis (RA). The exact mechanism of AD in promoting joint destruction remains unclear. Osteopontin (OPN) is required for osteoclast recruitment. We hypothesized that AD exacerbates bone erosion by inducing OPN expression in synovial tissue. This study aimed to evaluate a novel role for AD in RA.

**Methods:**

The serum levels of AD and OPN were determined in 38 patients with RA, 40 patients with osteoarthritis (OA), and 20 healthy controls using enzyme-linked immunosorbent assay (ELISA). AD and OPN production were measured by double immunofluorescence in RA and OA synovial tissue. Quantitative real-time PCR and immunofluorescence were used to evaluate the mRNA and protein expression levels of OPN in RA synovial fibroblasts (RASFs) and OA synovial fibroblasts after pre-incubation with AD, respectively. Migration of the RAW264.7 osteoclast precursor cell line was assessed using the Transwell migration assay and co-culture system. Bone destruction and osteoclastogenesis were assessed by immunohistochemical staining, microcomputed tomography and tartrate-resistant acid phosphatase (TRAP) staining in AD-treated collagen-induced arthritis (CIA) mice with or without OPN silencing. The expression levels of OPN and integrin α_v_β_3_ in the ankle joint tissues of the mice were examined by double immunofluorescence.

**Results:**

Our results indicated that the AD and OPN expression levels increased noticeably and were associated with each other in the RA serum. The AD distribution was coincident with that of OPN in the RA synovial tissue. AD stimulation of RASFs increased OPN production in a dose-dependent manner. AD-treated RASFs promoted RAW264.7 cell migration, and the effect was blocked with a specific antibody against OPN. Silencing of OPN using lentiviral-OPN short hairpin RNA reduced the number of TRAP-positive osteoclasts and the extent of bone erosion in the AD-treated CIA mice. When bound to integrin α_v_β_3_, OPN functions as a mediator of AD and osteoclasts.

**Conclusions:**

Our study provides new evidence of AD involvement in bone erosion. AD induces the expression of OPN, which recruits osteoclasts and initiates bone erosion. These data highlight AD as a novel target for RA treatment.

**Electronic supplementary material:**

The online version of this article (10.1186/s13075-018-1526-y) contains supplementary material, which is available to authorized users.

## Background

Rheumatoid arthritis (RA) is an autoimmune disease that is characterized by chronic synovial inflammation, joint destruction, progressive disability, systemic complications and socioeconomic costs [1, 2]. Articular bone erosion is a hallmark of RA that is linked to disease severity and poor functional outcomes [3]. Current intensive synthetic and biologic disease-modifying therapies sometimes fail or produce only partial responses in a subset of patients with RA, suggesting that there are additional pathways during disease progression. Therefore, the search for novel targets for RA therapy remains challenging.

Although the precise aetiology of RA remains elusive, substantial evidence has suggested that T cells, B cells and the complex interaction of multiple pro-inflammatory cytokines play critical roles in the pathophysiology of RA [3, 4]. Adipose tissue is a ubiquitous tissue, and accumulative evidence shows that the dominant cell type in adipose tissue, the adipocyte, can synthesize and release pro-inflammatory molecules, complement factors, signalling molecules, growth factors and adhesion molecules, thus contributing to a wide spectrum of diseases that include not only cardiovascular and metabolic complications but also inflammation-related and immune-related disorders [5, 6].

Adiponectin (AD), a 28–30 kDa collagen-like protein that comprises 244 amino acids, and is the most abundant adipokine in circulation that is secreted by adipocytes. AD is a multimeric protein that exists in multiple isoforms and has different biological activities. Additionally, AD has structural homology with collagen VIII, collagen X, and complement factor C1q. Elevated AD in synovial fluid in RA compared to synovial fluid in osteoarthritis (OA) indicates a pro-inflammatory role for AD in arthritis [7]. Furthermore, AD can promote the production of various inflammatory cytokines, including interleukin-6, interleukin-17 and TNF-α, in patients with RA and in a collagen-induced arthritis (CIA) mouse model. Moreover, AD influences RA bone remodelling through alterations in osteoblasts and osteoclasts [8]. Interestingly, recent clinical studies have suggested that the serum levels of AD are linked to the radiographic progression of RA, implying that AD might participate in bone erosion in RA [9]. Our previous study has demonstrated that AD is highly expressed in the inflamed synovial joint tissue and correlates well with progressive bone erosion in patients with RA [10]. Local AD injections into the joints of CIA mice results in early onset of arthritis symptoms, serious synovial hyperplasia, bone destruction and osteoporosis, compared with non-AD-treated mice [11]. However, the underlying mechanisms remain unclear.

Osteopontin (OPN), which is also known as early T lymphocyte activation 1, is a 34 kDa protein that is synthesized in various tissues and cells with pleiotropic properties. OPN is present in the extracellular matrix of mineralized tissue and in the extracellular fluids at sites of inflammation [12]. OPN is important for synovitis, cell migration and T helper 17 (Th17) cell differentiation [13–15]. The primary effects of OPN in bone tissue include osteoclast differentiation and activation [16]. Additionally, OPN is an important mediator of the immune response because it interacts with various cell-surface receptors, including multiple integrins [17]. OPN may promote osteoclast-mediated bone resorption by binding to its receptor, integrin α_v_β_3_, during arthritis. Binding of OPN to these cell-surface receptors stimulates cell adhesion, cell migration and other specific cell-signalling functions [18]. Interestingly, OPN deficiency prevents joint swelling, joint surface destruction and proteoglycan loss in the articular joint cartilage of CIA mice [19].

We conducted this study to identify a previously unrecognized regulatory role for AD in OPN expression during the pathological progression of RA. We hypothesized that AD could promote OPN expression in synovial tissue, linking to osteoclast anchoring to the bone surface and initiation of bone erosion. Therefore, we investigated the following: (1) the correlation between AD and OPN expression in the serum and synovial tissue of patients with RA, (2) the regulatory effect of AD on OPN expression and osteoclast precursor migration in synovial fibroblasts and (3) the effect of AD and OPN on disease progression, osteoclastogenesis and bone erosion in CIA mice. This study provides insights into the mechanism of AD functions and reveals a potential therapeutic target for arthritis treatments.

## Methods

### Reagents

Recombinant mouse and human AD were acquired from Peprotech (Rocky Hill, NJ, USA). Lentiviral particles carrying the OPN short hairpin RNA (Lenti-shOPN) were purchased from Genechem (Shanghai, China). The TRIzol reagent and SYBR Green I stain were obtained from Invitrogen (Carlsbad, CA, USA). The PrimerScript^TM^RT reagent kit was obtained from TaKaRa (Dalian, China). The Power SYBR Green PCR Master Mix was obtained from Applied Biosystems (Carlsbad, CA, USA). Mouse anti-AD monoclonal antibody (mAb), rabbit anti-OPN polyclonal Ab (pAb) and mouse anti-integrin α_v_β_3_ mAb were obtained from Abcam (Abcam, Cambridge, UK). Donkey anti-mouse IgG-R and goat anti-rabbit IgG/TRITC were obtained from Santa Cruz Biotechnology (Santa Cruz, CA, USA). Alexa Fluor 488 AffiniPure donkey anti-rabbit IgG, peroxidase-conjugated sheep anti-rabbit secondary antibody (Ab) and peroxidase-conjugated sheep anti-mouse secondary Ab were obtained from Jackson Immunoresearch (West Grove, PA, USA). Tissue culture reagents including Dulbecco’s modified Eagle’s medium (DMEM) and fetal bovine serum (FBS) were purchased from Gibco (Carlsbad, CA, USA). The leukocyte acid phosphatase kit for tartrate-resistant acid phosphatase (TRAP) staining was obtained from Sigma-Aldrich (St. Louis, MO, USA). The human AD and OPN enzyme-linked immunosorbent assay (ELISA) kits and anti-human OPN Ab were obtained from eBioscience (Los Angeles, CA, USA). The lipopolysaccharide (LPS) ELISA kit was from ELBAS (Shanghai, China).

### Patients and samples

Patients with RA and OA and the healthy controls (HC) were recruited randomly from the First Affiliated Hospital of Nanjing Medical University. Blood samples were gathered from 38 patients with RA, 40 patients with OA, and 20 HC. Synovial tissue was obtained from three patients with RA and three with OA who had undergone therapeutic synovectomy. The classification of RA fulfilled 2010 American College of Rheumatology (ACR)/European League Against Rheumatism (EULAR) classification criteria [20]. OA diagnosis was determined by clinician assessment according to the criteria [21]. This study was approved by the Ethics Committee at the First Affiliated Hospital of Nanjing Medical University, and all donors signed informed consent forms.

Blood samples were collected from the peripheral veins, centrifuged and stored at −80 °C. The synovial tissue was prepared for cell culture experiments or stored in a buffered 4% paraformaldehyde fixative for immunohistological analysis.

### ELISA

The concentration of LPS in the AD and the serum levels of AD and OPN for RA, OA, and HC were measured using ELISA kits according to the manufacturer’s instructions. All measurements were performed in duplicate.

### Cell cultures

Primary RA synovial fibroblasts (RASFs) were isolated from the synovial tissue of patients with RA as previously described [22]. Primary OA synovial fibroblasts (OASFs) were isolated from the synovial tissue of patients with OA. Briefly, the synovial tissue was minced and digested with 1% collagenase II at 37 °C. RASFs or OASFs were cultured in DMEM supplemented with 10% FBS, 100 U/mL penicillin and 100 μg/mL streptomycin at 37 °C with 5% CO_2_.

### Quantitative real-time PCR

Total RNA was extracted using TRIzol (Invitrogen Inc., Carlsbad, CA, USA) from RASFs or OASFs that were incubated with different doses of AD, and the RNA was reverse-transcribed using the PrimeScript RT-PCR Kit according to the manufacturer’s instructions (TaKaRa). Quantitative real-time PCR was conducted using the Applied Biosystems 7900HT Instrument (Applied Biosystems, Carlsbad, CA, USA) and the SYBR Green PCR Master Mix. The primer sequences for the genes were as follows: OPN, sense 5’-GAAGTTTCGCAGACCTGACAT-3’, antisense 5’-GTATGCACCATTCAACTCCTCG-3’; GAPDH, sense 5’-TGACTTCAACAGCGACACCCA-3’, antisense 5’-CACCCTGTTGCTGTAGCCAAA-3’. The cycling conditions included an initial denaturation procedure at 95 °C for 10 min, followed by 40 cycles of 95 °C for 15 sec and 60 °C for 1 min. Relative gene expression was determined by 2^-ΔΔCt^.

### RASFs or OASFs and RAW264.7 co-culture and Transwell migration assay

RASFs or OASFs were incubated with the AD or the AD and anti-OPN Abs in 24-well flat-bottom plates for 72 h. RAW264.7 cells (1.5 × 10^4^) were added to the upper chambers of the Transwell inserts (8-μm pores, 6.5-mm polycarbonate membranes, Costar, Corning, NY, USA) and co-cultured with the RASFs or OASFs for 24 h at 37 °C (5% CO_2_). After 24 h, the RAW264.7 cells in the upper compartment were gently wiped away with a cotton swab to remove the unmigrated cells, and the migrated cells on the undersides of the membranes in the Transwells were fixed with paraformaldehyde and stained with crystal violet. The bound dye was released with 30% glacial acetic acid, and the optical density of the solution was measured at 570 nm using an enzyme immunosorbent assay reader.

### Collagen-induced arthritis model in vivo

Male DBA/1 J mice (aged 8–10 weeks) were purchased from Shanghai Laboratory Animal Center, Chinese Academy of Science. The mice were maintained under specific pathogen-free conditions and fed the standard mouse chow and water ad libitum. CIA mice were induced as previously described [11]. Briefly, 100 μg of bovine collagen type II (CII; Chondrex, Redmond, WA, USA) that was dissolved in 0.05 M acetic acid was emulsified with an equal volume of Freund’s complete adjuvant (Difco, Detroit, MI, USA) and administered intradermally at the base of tail into DBA/1 J mice. On day 21 after the initial immunization, a booster emulsion prepared with CII and Freund’s incomplete adjuvant (Difco) was injected intradermally near the primary injection site. Groups of 10 CIA mice were intra-articularly injected with 10 μL of AD (1 μg/μL) into the knee joints on days 17, 20 and 23 after the first CII immunization, and the other knees were treated with an equivalent volume of PBS as the controls. A group of five CIA AD-treated mice were additionally injected with 1 × 10^7^ TU Lenti-shOPN on the day of the second CII immunization.

Mice were monitored daily after the 2nd CII immunization in a blinded manner to determine the severity of arthritis, as previously described [23]. Briefly, all four mouse limbs mice were evaluated and scored from 0 to 4 according to the following scale: 0 = no swelling; 1 = slight swelling and erythema that was confined to either the ankle or mid-foot region; 2 = slight swelling that extended from the ankle to the mid-foot region; 3 = moderate swelling from the ankle to the metatarsal joints; and 4 = severe swelling in the ankle, foot and digits.

Upon sacrifice on day 45, the paws and knee joints of the mice were removed immediately and fixed in 4% paraformaldehyde for microcomputed tomography (microCT) analysis, using a microCT scanner (Skyscan 1176; Bruker, Kontich, Belgium) at 9-μm resolution. For verification of bone destruction, 3-dimensional models were reconstructed and analysed using the Skyscan software.

### Immunohistochemical analysis

For the haematoxylin and eosin (H&E) staining and immunohistochemical analysis, the ankles or paws were isolated from euthanized mice, fixed in 4% buffered paraformaldehyde, decalcified in 50 mM ethylene diamine tetraacetic acid, embedded in paraffin, and serially sectioned. The tissues were then sectioned into 3-μm slices, deparaffinized in xylene, rehydrated using a series of ethanol concentrations and stained with H&E. After inactivation of the endogenous peroxidase, sections were sequentially blocked with 5% bovine serum album for 30 min at room temperature and incubated with rabbit anti-OPN pAb at 4 °C overnight in a humidified chamber. After washing, sections were additionally incubated with peroxidase-conjugated goat anti-rabbit secondary Ab for 1 h at room temperature. The reactions were followed using a 3,3-diaminobenzidine (DAB) substrate kit, with haematoxylin as the counterstain. To analyse the osteoclasts in the joint tissues, each joint section was processed using the TRAP kit.

### Immunofluorescence staining

For cell immunofluorescence staining, RASFs or OASFs that were incubated with AD at various concentrations were fixed in 4% paraformaldehyde for 10 min and permeabilized with 0.3% Triton X-100 in PBS for 5 min. The cells were incubated with rabbit anti-OPN pAb overnight at 4 °C, washed and further incubated with goat anti-rabbit IgG/TRITC for 1 h at room temperature. Finally, the cells were incubated with a 4',6-diamidino-2-phenylindole (DAPI) staining solution for 1–2 min after washing and examined by fluorescence microscopy (Nikon, Japan). OPN positivity was indicated in red, and the nuclei were indicated in blue.

For double immunofluorescence labelling of the tissues, the murine joint tissue serial sections were incubated with a mixture of primary antibodies (rabbit anti-OPN pAb and mouse anti-Integrin α_v_β_3_ mAb) at 4 °C overnight. Slides were then incubated with a mixture of donkey anti-mouse IgG-R, Alexa Fluor 488 AffiniPure Donkey Anti-Rabbit IgG and DAPI for 1 h. Images were collected under a fluorescence microscope and processed digitally. AD positivity was indicated in red, OPN positivity was indicated in green, and the nuclei were indicated in blue. The paraffin-embedded human synovial tissue was serially sectioned, dewaxed, rehydrated and stained following our published procedures [24]. The sections were stained with a mouse anti-AD mAb and rabbit anti-OPN pAb, followed by DyLight™488-conjugated donkey anti-mouse IgG, donkey anti-rabbit IgG-R and DAPI.

### Statistical analysis

Statistical analyses were performed using the SPSS software (SPSS, Inc, Chicago, IL, USA), and all figures were performed using the GraphPad Prism 6.0 software (GraphPad Software, La Jolla, CA, USA). Data were expressed as the mean ± SD. Differences between the two groups were analysed using the non-paired two-tailed Student *t* test followed by the Bonferroni correction. The correlation between the AD and OPN levels in the patients with RA was tested using Spearman’s rank correlation coefficient. For all experiments, *p* < 0.05 was considered statistically significant.

## Results

### AD is correlated with OPN in serum and synovial tissue of patients with RA

**Fig. 1:**
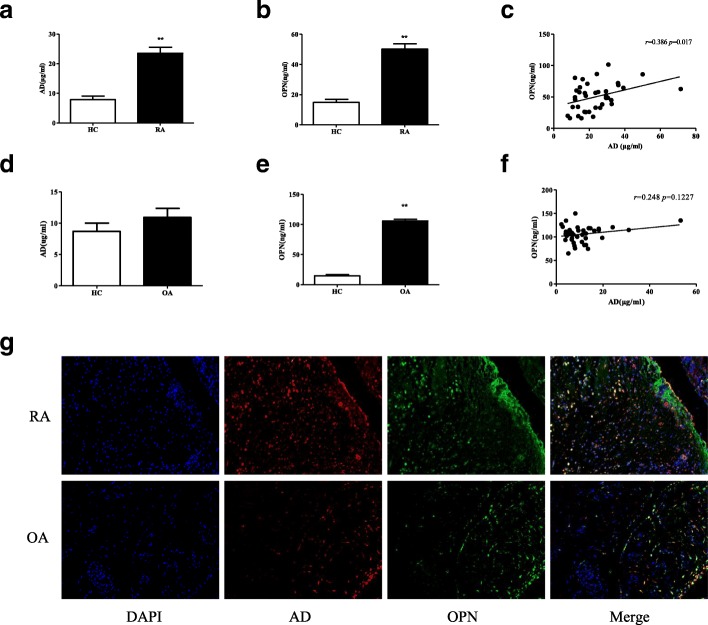
Correlation between adiponectin (AD) and osteopontin (OPN) expression in serum and synovial tissue of patients with rheumatoid arthritis (RA). **a** Serum AD levels were determined in patients with RA (*n* = 38) and in healthy controls (HC) (*n* = 20) by ELISA. **b** Serum OPN levels were measured in patients with RA (*n* = 38) and HC (*n* = 20) by ELISA. **c** The positive relationship between serum AD and OPN levels in patients with RA. **d** Serum AD levels were examined in patients with osteoarthritis (OA) (*n* = 40) and in HC (*n* = 20) by ELISA. **e** Serum OPN levels were tested in patients with OA (*n* = 40) and in HC (*n* = 20) by ELISA. **f** No relationship is observed between serum AD and OPN levels in patients with OA. **g** Double immunofluorescence analysis of AD and OPN expression in RA and OA synovial tissue (*n* = 3). Each section was merged with 4’,6-diamidino-2-phenylindole (DAPI) (magnifications: ×20). Bars show the mean ± SD. The degree of linearity between the two variables was compared using Spearman’s correlation test (two-tailed). **p < 0.01 versus control (**a**, **b** and **e**)

To examine the effects of AD and OPN during the development of RA, we first assessed the relationship between AD and OPN expression in patients with RA. The AD expression levels in serum from the patients with RA were significantly higher than in serum from the HC (Fig. 1a), and the increased AD expression was accompanied by noticeably elevated OPN in serum from the patients with RA (Fig. 1b). There was positive correlation between elevated AD and OPN in serum from the patients with RA (*p* = 0.017, *r* = 0.386) (Fig. 1c). There were no differences in AD expression levels between patients with OA and the HC (Fig. 1d). The levels of OPN were higher in patients with OA than in the HC (Fig. 1e). There was no relationship between AD and OPN in patients with OA (Fig. 1f). The double immunofluorescence analysis indicated marked AD and OPN co-staining in the RA synovial tissue compared with tissue from the patients with OA (Fig. 1g). Medication records of RA patients for the serum samples were summarized. Medication records of RA patients for the synovial tissue samples were summarized. These data suggest that AD and OPN increase in the RA serum and synovial tissue and that AD and OPN expression correlate with each other.

### AD promotes OPN production in RASFs

**Fig. 2:**
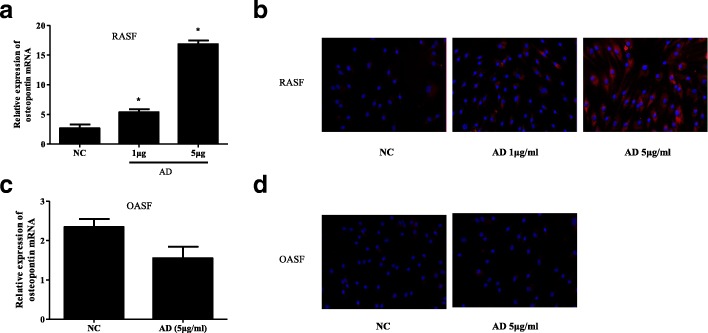
Concentration-dependent increase in osteopontin (OPN) production by adiponectin (AD). **a** Human rheumatoid arthritis synovial fibroblasts (RASFs) were incubated with AD for various doses, and OPN mRNA expression was examined after 72 h by quantitative real-time PCR (n = 3). **b** OPN expression in human RASFs pretreated with AD for different concentrations was determined after 72 h by immunofluorescence staining. **c** Human osteoarthritis synovial fibroblasts (OASFs) were incubated with AD, and OPN mRNA expression was examined after 72 h by quantitative real-time PCR (n = 3). **d** OPN expression in human OASFs pretreated with AD was determined after 72 h by immunofluorescence staining. OPN-positive cells were stained red (magnification ×20). Bars show the mean ± SD (*p < 0.05)

To further verify the relationship between AD and OPN, we examined the effect of AD on OPN expression in RASFs. OPN mRNA production was significantly increased in a dose-dependent manner in RASFs in response to various dosages (1 μg/mL and 5 μg/mL) of AD for 72 h (Fig. 2a). The immunofluorescence analysis showed that RASFs that were stimulated with AD for 72 h also exhibited an increase in OPN expression in a concentration-dependent manner (Fig. 2b). We found that AD had no effect on OPN mRNA production and protein levels in OASFs when OASFs was incubated with AD (5 μg/mL) (Fig. 2c, d). These results imply that AD induces upregulation of the OPN mRNA and protein levels in RASFs.

### AD promotes RAW264.7 migration by enhancing OPN production in RASFs

**Fig. 3:**
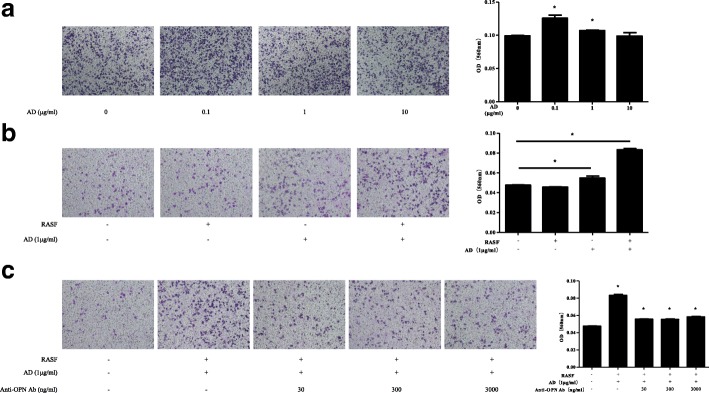
RAW264.7 migration assay by crystal violet staining. **a** Rheumatoid arthritis synovial fibroblasts (RASFs) were incubated with various concentrations of adiponectin (AD) (0.1, 1 and 10 μg/mL) in 24-well flat-bottom plates for 72 h. RAW264.7 cells were added to Transwell inserts and co-cultured with the aforementioned RASFs for 24 h. The effect of RAW264.7 migration was examined (*n* = 3). **b** Culture medium or RASFs were incubated with or without AD (1 μg/mL) in 24-well flat-bottom plates for 72 h. RAW264.7 cells were added to Transwell inserts and co-cultured with the above culture medium or RASFs for 24 h. The effect of RAW264.7 migration was measured (*n* = 3). **c** RASFs were incubated with AD (1 μg/mL) and a serial dilution of OPN (30, 300 and 3000 ng/mL) in 24-well flat-bottom plates for 72 h. RAW264.7 cells were added to Transwell inserts and co-cultured with the above RASFs for 24 h. The effect of RAW264.7 migration was investigated (*n* = 3). Bars show the mean ± SD; *p < 0.05 vs control. The experiment was repeated three times and representative pictures are shown. Ab, antibodies; OD, optical density

Next, we investigated the functional role of AD-upregulated OPN. We observed that incubation of RASFs with 0.1 μg/mL or 1 μg/mL AD in the lower compartments of the co-culture system significantly increased RAW264.7 migration through the Transwell microporous membranes, whereas incubation of RASFs with 10 μg/mL AD was not statistically significant (Fig. 3a). RASFs alone could not promote RAW264.7 migration without the AD pre-treatment. However, RASFs that were pre-incubated with AD (1 μg/mL) significantly increased RAW264.7 migration in the co-culture system (Fig. 3b). As controls, OASFs pre-incubated with AD (1 μg/mL) did not significantly increase RAW264.7 migration in the co-culture system (see Additional file 1: Figure S1). Furthermore, RASF-mediated RAW264.7 migration was reduced with the anti-OPN Abs (30, 300 and 3000 ng/mL) in the co-culture system (Fig. 3c). The concentration of LPS in AD was detected and no differences were found between the blank culture medium and AD used in the assays (see Additional file 2: Figure S2). Our results demonstrate that AD-treated RASFs promote migration of RAW264.7 osteoclast precursor cells by upregulating OPN expression.

### Knockdown of OPN decreases synovial inflammation and bone erosion in vivo

**Fig. 4:**
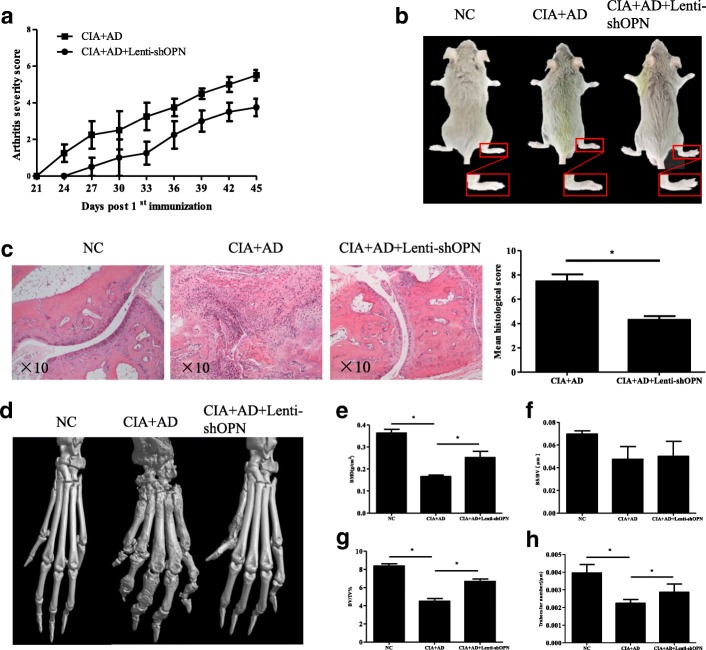
Lenti-shOPN injection significantly attenuated synovial inflammation and bone erosion in mice with adiponectin (AD)-treated collagen-induced arthritis (CIA). **a** Arthritis severity scores and incidence of CIA development in AD-treated CIA mice were recorded daily after 2nd collagen type II (CII) immunization (n = 5). **b** Representative photographs of AD-treated CIA mice with or without Lenti-shOPN. **c** Histologic sections of ankle joints were stained with H&E in the indicated groups and values of histopathological scores are shown. Bars show the mean ± SD (*p < 0.05). **d** Representative three-dimensional renditions of the ankles and paws scanned by microcomputed tomography (microCT). **e**-**h** Quantification of bone mineral density (**e**), ratio between bone surface and bone volume (**f**), ratio between bone volume and tissue volume (**g**) and trabecular number (**h**) was calculated. Values were analyzed using microCT Skyscan software. Bars show the mean ± SD (*p < 0.05)

To further verify the role of OPN *in vivo*, we evaluated the effect of shRNA-mediated OPN knockdown in slowing AD-mediated disease progression. AD-treated mice had more serious arthritis and bone erosion. However, local Lenti-shOPN injections in the AD-treated CIA mice resulted in delayed onset of arthritis and low arthritis scores relative to the control AD-treated CIA mice (Fig. 4a, b). Furthermore, the histopathological analysis revealed less pronounced synovial hyperplasia and bone erosion in the AD-treated CIA mice after the local OPN knockdown than in the control AD-treated CIA mice (Fig. 4c). The microCT examination showed less severe bone damage in the periarticular bones of the paws and ankles in the AD-treated CIA mice after the Lenti-shOPN injection than in the control AD-treated CIA mice (Fig. 4d). Mice with AD-treated CIA had significant reduction in bone mineral density (BMD) at the ankle joint (Fig. 4e). There were no differences in the ratio between bone surface and bone volume among the NC group, the AD-treated CIA with or without Lenti-shOPN administration group (Fig. 4f). Mice with AD-treated CIA had significant reduction in the ratio between bone volume and tissue volume at the ankle joint (Fig. 4g). Mice with AD-treated CIA had significant reduction in trabecular number (Fig. 4h). In contrast, AD-treated CIA mice with Lenti-shOPN administration had higher BMD, ratio between bone volume and tissue volume and trabecular number at the ankle joints, as compared with the AD-treated CIA group (Fig. 4e, g, and h). Taken together, our results confirm that local OPN suppression prevents synovial inflammation and joint erosion progression in AD-treated mice. These results are consistent with our observations of disease outcome in vitro.

### Knockdown of OPN reduces the number of AD-induced TRAP-positive osteoclasts in vivo

**Fig. 5:**
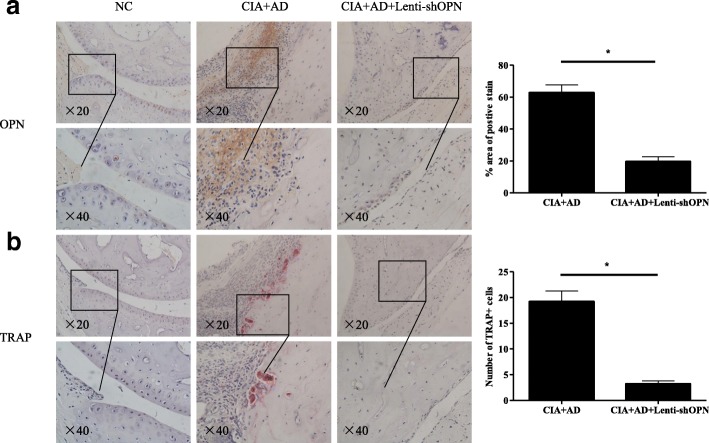
Reduced expression of osteopontin (OPN) and tartrate-resistant acid phosphatase (TRAP)-positive osteoclasts in adiponectin (AD)-treated collagen-induced arthritis (CIA) mice with Lenti-shOPN administration. **a** Serially sectioned immunohistochemical staining of OPN in the ankle joint tissue of AD-treated CIA mice with or without Lenti-shOPN on day 45 post the 1st collagen type II (CII) immunization (magnification ×20 and ×40). OPN-expressing cells were stained with intense brown color. **b** Serially sectioned immunohistochemical TRAP staining in the ankle joint tissue of AD-treated CIA mice with or without Lenti-shOPN on day 45 post the 1st CII immunization (magnification ×20 and ×40). TRAP-positive osteoclasts were stained red (n = 5). The results shown are representative of one of three independent experiments. Bars show the mean ± SD (*p < 0.05)

To further assess the relationship between AD, OPN and osteoclasts in vivo, we performed immunohistochemical analysis of serial sections of the inflamed ankle joint tissues. The number of OPN-positive cells was significantly increased in the ankle joints of the AD-treated CIA mice and noticeably decreased after the local OPN knockdown (Fig. 5a). Consistent with OPN expression, the number of TRAP-positive osteoclasts was also dramatically elevated in the AD-treated CIA mice but markedly diminished after the local Lenti-shOPN knockdown (Fig. 5b). These data indicate that AD directly induces OPN expression and osteoclast production.

### Involvement of integrin α_v_β_3_ in AD-induced OPN expression in vivo

**Fig. 6:**
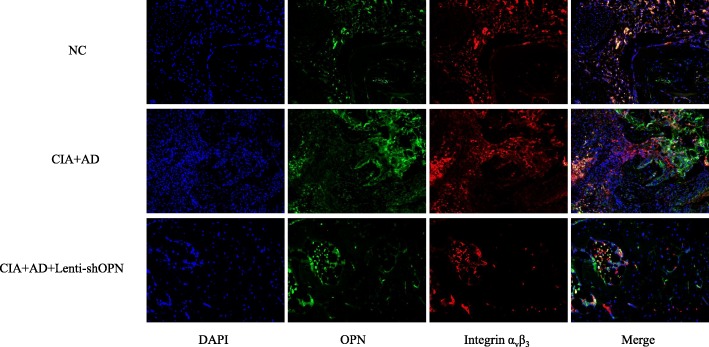
Co-localization of osteopontin (OPN) and integrin α_v_β_3_ in adiponectin (AD)-treated collagen-induced arthritis (CIA) mice administered or not administered Lenti-shOPN. Double immunofluorescence analysis of OPN and integrin α_v_β_3_ expression in the ankle joint tissue of AD-treated CIA mice with or without Lenti-shOPN injection on day 45 post the 1st collagen type II (CII) immunization (*n* = 5). Each section was merged with 4’,6-diamidino-2-phenylindole (DAPI) (magnification ×20)

OPN may bind to its receptor, integrin α_v_β_3_, to mediate bone resorption by osteoclasts in arthritis. Therefore, we confirmed the expression and colocalization of OPN and integrin α_v_β_3_ in the ankle joint tissues of the animal models. OPN expression in the cells was indicated in green, integrin α_v_β_3_ positivity was indicated in red, and OPN plus integrin α_v_β_3_ positivity was indicated in yellow. As expected, OPN and integrin α_v_β_3_ were strongly decreased in the ankle joints of the AD-treated CIA mice with Lenti-shOPN compared with their levels in the AD-treated CIA mice (Fig. 6). Taken together, our results show that integrin α_v_β_3_ is detected coincidently with OPN, which further confirms that the effect of AD-induced OPN on osteoclast precursor migration and progression of bone erosion is mediated through integrin α_v_β_3_.

## Discussion

This study provides new evidence of the novel role of AD in bone erosion and the underlying mechanism of AD-mediated bone erosion in RA. Our most recent study demonstrated that AD correlates closely with progressive bone erosion in patients with RA [10] and that AD aggravates the process by enhancing the Th17 cell response and receptor activator of nuclear factor-κB ligand (RANKL) expression in CIA mice [11]. However, AD could inhibit osteoclast formation and differentiation [25, 26]. These contradictory findings prompted us to reveal the real role of AD on osteoclasts. Our study indicates positive correlation between OPN and AD expression in patients with RA. Furthermore, OPN expression is upregulated by AD in RASFs, which results in the enhanced activity of osteoclast precursor migration and osteoclastogenesis, thus contributing to articular destruction. This effect can be blocked by inhibiting OPN, which confirms the role of OPN in the migration and osteoclastogenic process.

We observed that AD and OPN were both highly expressed in serum and synovial tissue from the patients with RA. The physiological blood AD concentration is 1–10 μg/mL. Recent studies have shown that a marked increase in plasma AD levels is evident in patients with RA [27] and that the increase is associated with high disease activity [28]. Furthermore, plasma OPN levels are significantly decreased after treatment and may reflect inflammatory bone erosion [29]. We have demonstrated that AD and OPN are positively related to each other in patients with RA. Their correlation is evident in the mechanisms of the renin angiotensin aldosterone system and inflammation [30]. Moreover, our data indicate that AD can upregulate OPN mRNA and protein expression in RASFs. Pretreatment of RASFs with AD significantly increases the expression of cytokines, chemokines and matrix metalloproteinases (MMPs), suggesting that AD has a broad spectrum of pro-inflammatory properties in RA synovitis. The effects of AD on RASFs are strongly selective, and AD functions by inducing IL-6 and MMP-1 via the p38 mitogen-activated protein kinase (MAPK), activated protein kinase (AMPK) and NF-κB signalling pathways [31]. OPN is produced by RASFs in the synovial lining and at sites of cartilage invasion. OPN mediates the attachment of synovial T cells to cartilage and contributes to matrix degradation by stimulating the secretion of collagenase 1 in articular chondrocytes [32]. Endogenous production of OPN in RASFs is attributable to increased production of IL-17 in T cells and plays a critical role in Th17 differentiation [13]. Although, the underlying mechanism of AD-induced upregulation of OPN requires further study, our results indicate that both AD and OPN are involved in the development of RA.

Notably, our study presents valid evidence that OPN is an important mediator of AD-induced bone erosion. The potency of OPN towards AD and osteoclasts is demonstrated by the AD-associated enhancement of OPN expression that results in osteoclast precursor migration and osteoclast formation. Inhibition of OPN reduces migration and osteoclastogenesis and prevents the progression of bone destruction. Chemokines, such as IL-8 and monocyte chemoattractant protein 1 (MCP-1), which are secreted by RASFs upon AD stimulation, increase the migration of lymphocytes and RASFs [33]. OPN selectively induces the expression of pro-inflammatory cytokines and chemokines, such as IL-1 and IL-8, which promote migration and the recruitment of inflammatory cells. The effect of OPN on inflammatory cell migration is mediated through MCP-1 and macrophage inflammatory protein-1β (MIP-1β) via the NF-κB and MAPK signalling pathways, which are involved in the activation of inhibitor of NF-κB kinase-β (IKKβ), p38, and c-Jun N-terminal kinase (JNK) in RA CD14+ monocytes, and the OPN effect can be blocked with an anti-OPN Ab [34]. Additionally, OPN enhances monocyte migration via the Syk/PI3K/Akt signalling pathway in RA [14]. Osteoclastic recruitment is a complex process that is closely related to migratory factors and the microenvironment. Osteoclast precursors systemically circulate and migrate to the bone surfaces that are targeted for resorption. Chemokines, such as CXCL12 and CX_3_CL1, promote the chemotactic recruitment, development, and survival of osteoclast precursors [35, 36].

OPN, an anchor of osteoclasts to bone [37], is an active factor that is involved in the recruitment of osteoclasts. OPN functions as a positive regulator through the RANK/RANKL/osteoprotegerin (OPG) system during the osteoclastogenic process of arthritis. RANKL binds to its receptor, RANK, on the osteoclast precursors, which leads to the expression of various osteoclast genes, including TRAP, cathepsin K, calcitonin receptor, integrin α_v_β_3_ and MMP-9. Inhibition of OPN may be an effective treatment for bone destruction that functions not only by inhibiting osteoclast activation but also by preventing osteoclast differentiation [16]. Taken together, our results confirm that AD may recruit osteoclasts through OPN in vitro and in vivo, which directly leads to bone erosion.

The action mode of the osteoclast, which is a key player in joint and bone destruction, consists of several processes that include migration, differentiation and bone resorption. The increased bone resorption in osteoporosis and RA is linked to the facilitation of osteoclast differentiation and activation. Patients with RA experience systemic osteoporosis and the effects of RA on periarticular bone loss were replicated in our CIA animal model. Inhibition of OPN may be effective in restoring BMD, the ratio between bone volume and tissue volume and trabecular number at the ankle joints. Osteoclast attachment to the bone surface occurs through integrins, particularly integrin α_v_β_3_ [38]. OPN contains a functional arginine-glycine-aspartate (RGD)-binding motif that is specifically recognized by integrin α_v_β_3_. Integrin α_v_β_3_, which binds to OPN with high affinity and transduces signals from the outside of the cell to the interior, increases the bone resorptive capacity of osteoclasts by activating intracellular signalling pathways such as focal adhesion kinase (FAK) and c-Src [39, 40]. In our study, OPN expression was detected mainly at sites of activated osteoclasts in the CIA mice. Additionally, the integrin α_v_β_3_ distribution was coincident to that of OPN. Our results further explain the molecular mechanism by which OPN plays crucial roles in osteoclast processes when bound to integrin α_v_β_3._

## Conclusions

In summary, we have presented new evidence that AD mediates bone erosion by inducing the production of OPN, which recruits osteoclasts to the bone surface and initiates bone erosion. Further investigations of other biological functions and the molecular mechanism of AD-induced OPN expression will provide a more complete understanding of the pathological functions of AD in RA development.

## Additional files

Additional file 1: Figure S1. RAW264.7 migration assay by crystal violet staining. OASFs or RASFs were incubated with or without AD (1 μg/mL) in 24-well flat-bottom plates for 72 h. RAW264.7 cells were added to Transwell inserts and co-cultured with the above OASFs or RASFs for 24 h. The effect of RAW264.7 migration was measured (n = 3). Bars show the mean ± SD; *p < 0.05 vs control. The experiment was repeated three times, and representative pictures are shown. (PDF 459 kb)

Additional file 2: Figure S2. Detection of LPS concentration in AD. LPS concentration was measured by ELISA. Bars show the mean ± SD; *p < 0.05 vs control. (PDF 157 kb)

## Abbreviations

Ab: Antibodies
ACR: American College of Rheumatology
AD: Adiponectin
BMD: Bone mineral density
C II: Collagen type II
CIA: Collagen-induced arthritis
DAPI: 4',6-Diamidino-2-phenylindole
DMEM: Dulbecco’s modified Eagle’s medium
ELISA: Enzyme-linked immunosorbent assay
EULAR: European League Against Rheumatism
FBS: Fetal bovine serum
H&E: Haematoxylin and eosin
HC: Healthy controls
IL: Interleukin
kDa: KiloDalton
LPS: Lipopolysaccharide
mAb: Monoclonal antibody
microCT: Microcomputed tomography
MMPs: Matrix metalloproteinases
NC: Normal controls
OA: Osteoarthritis
OASFs: Osteoarthritis synovial fibroblasts
OPN: Osteopontin
pAb: Polyclonal antibody
PBS: Phosphate-buffered saline
RA: Rheumatoid arthritis
RANKL: Receptor activator of nuclear factor-κB ligand
RASFs: Rheumatoid arthritis synovial fibroblasts
Sh: Short hairpin
Th17: T helper 17
TNF: Tumour necrosis factor
TRAP: Tartrate-resistant acid phosphatase
