# Correction to: Adiponectin aggravates bone erosion by promoting osteopontin production in synovial tissue of rheumatoid arthritis

**DOI:** 10.1186/s13075-018-1641-9

**Published:** 2018-06-13

**Authors:** Jie Qian, Lingxiao Xu, Xiaoxuan Sun, Yani Wang, Wenhua Xuan, Qian Zhang, Pengfei Zhao, Qin Wu, Rui Liu, Nan Che, Fang Wang, Wenfeng Tan, Miaojia Zhang

**Affiliations:** 1 Department of Rheumatology, The First Affiliated Hospital of Nanjing Medical University, 300 Guangzhou Road, Nanjing, 210029 China; 2 Department of Rheumatology, Affiliated Hospital of Nantong University, 20 Xisi Road, Nantong, 226001 China; 3 Department of Cardiology, The First Affiliated Hospital of Nanjing Medical University, 300 Guangzhou Road, Nanjing, 210029 China

## Correction

Unfortunately, after publication of this article [1], it was noticed that the panel for Fig. 4b was inadvertently obscured during the production process. The full, correct Fig. 4 can be seen below and the original article has been corrected to reflect this.

**Fig. 4:**
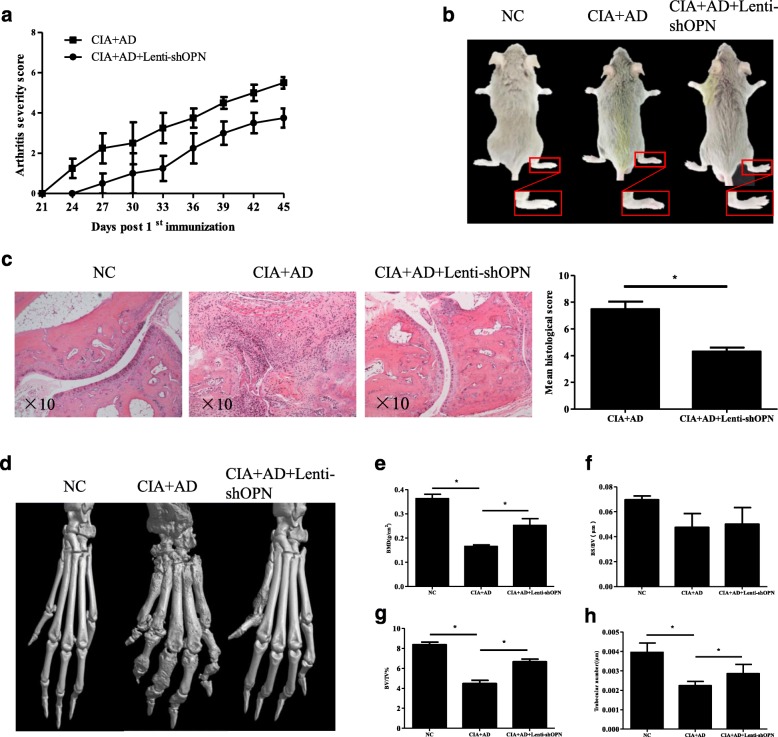
Lenti-shOPN injection significantly attenuated synovial inflammation and bone erosion in mice with adiponectin (AD)-treated collagen-induced arthritis (CIA). **a** Arthritis severity scores and incidence of CIA development in AD-treated CIA mice were recorded daily after 2nd collagen type II (CII) immunization (*n* = 5). **b** Representative photographs of AD-treated CIA mice with or without Lenti-shOPN. **c** Histologic sections of ankle joints were stained with H&E in the indicated groups and values of histopathological scores are shown. Bars show the mean ± SD (*p < 0.05). **d** Representative three-dimensional renditions of the ankles and paws scanned by microcomputed tomography (microCT). **e-h** Quantification of bone mineral density (**e**), ratio between bone surface and bone volume (**f**), ratio between bone volume and tissue volume (**g**) and trabecular number (**h**) was calculated. Values were analyzed using microCT Skyscan software. Bars show the mean ± SD (*p < 0.05)
