# Supplementary figures and images for: Adiponectin aggravates bone erosion by promoting osteopontin production in synovial tissue of rheumatoid arthritis
Source: Arthritis Res Ther. 2018 Feb 8;20:26. doi: 10.1186/s13075-018-1526-y (PMC5806355; doi:10.1186/s13075-018-1526-y)

Additional file 1: Figure S1

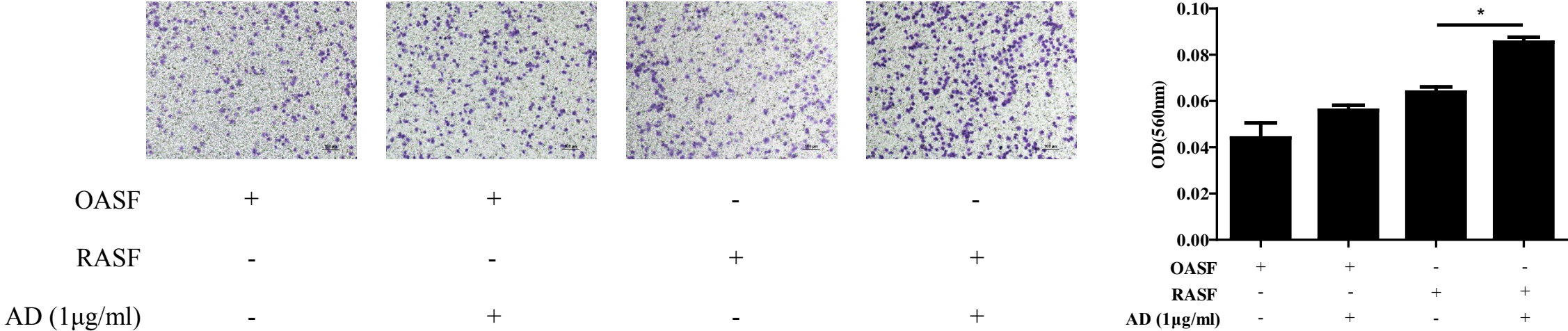

Supplement: Supplementary file 1 — RAW264.7 migration assay by crystal violet staining. OASFs or RASFs were incubated with or without AD (1 μg/mL) in 24-well flat-bottom plates for 72 h. RAW264.7 cells were added to Transwell inserts and co-cultured with the above OASFs or RASFs for 24 h. The effect of RAW264.7 migration was measured (n = 3). Bars show the mean ± SD; *p < 0.05 vs control. The experiment was repeated three times, and representative pictures are shown. (PDF 459 kb) [file 13075_2018_1526_MOESM1_ESM.pdf]

Additional file 2: Figure S2

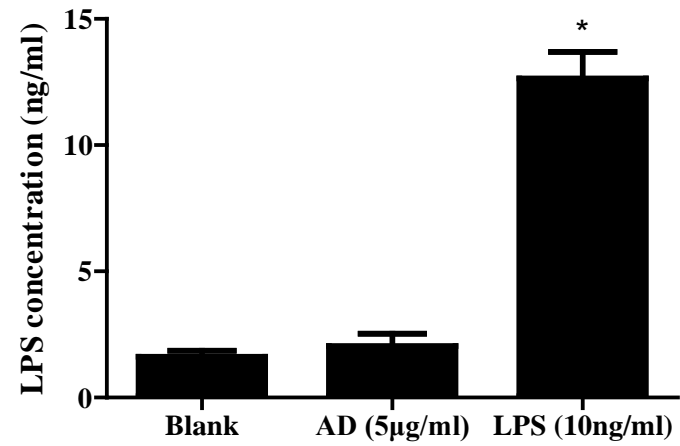

Supplement: Supplementary file 2 — Detection of LPS concentration in AD. LPS concentration was measured by ELISA. Bars show the mean ± SD; *p < 0.05 vs control. (PDF 157 kb) [file 13075_2018_1526_MOESM2_ESM.pdf]
